# Vagal blockade suppresses the phase I heart rate response but not the phase I cardiac output response at exercise onset in humans

**DOI:** 10.1007/s00421-021-04769-3

**Published:** 2021-08-14

**Authors:** Timothée Fontolliet, Aurélien Bringard, Alessandra Adami, Nazzareno Fagoni, Enrico Tam, Anna Taboni, Guido Ferretti

**Affiliations:** 1 Department of Basic Neurosciences, University of Geneva, Geneva, Switzerland; 2 Department of Anaesthesiology, Clinical Pharmacology, Intensive Care and Emergency Medicine, University of Geneva, Geneva, Switzerland; 3 Pulmunology Service, Geneva University Hospitals, Geneva, Switzerland; 4 Department of Kinesiology, University of Rhode Island, Kingston, RI USA; 5 AAT Brescia, Azienda Regionale Emergenza Urgenza (AREU), ASST Spedali Civili di Brescia, Brescia, Italy; 6 Department of Neurosciences, Biomedicine and Movement Sciences, University of Verona, Verona, Italy; 7 Department of Molecular and Translational Medicine, University of Brescia, Brescia, Italy

**Keywords:** Heart rate, Stroke volume, Cardiac output, Exercise transients, atropine

## Abstract

**Purpose:**

We tested the vagal withdrawal concept for heart rate (HR) and cardiac output (CO) kinetics upon moderate exercise onset, by analysing the effects of vagal blockade on cardiovascular kinetics in humans. We hypothesized that, under atropine, the *φ*_1_ amplitude (*A*_1_) for HR would reduce to nil, whereas the *A*_1_ for CO would still be positive, due to the sudden increase in stroke volume (SV) at exercise onset.

**Methods:**

On nine young non-smoking men, during 0–80 W exercise transients of 5-min duration on the cycle ergometer, preceded by 5-min rest, we continuously recorded HR, CO, SV and oxygen uptake ($\dot{V}$O_2_) upright and supine, in control condition and after full vagal blockade with atropine. Kinetics were analysed with the double exponential model, wherein we computed the amplitudes (*A*) and time constants (*τ*) of phase 1 (*φ*_1_) and phase 2 (*φ*_2_).

**Results:**

In atropine versus control, *A*_1_ for HR was strongly reduced and fell to 0 bpm in seven out of nine subjects for HR was practically suppressed by atropine in them. The *A*_1_ for CO was lower in atropine, but not reduced to nil. Thus, SV only determined *A*_1_ for CO in atropine. *A*_2_ did not differ between control and atropine. No effect on *τ*_1_ and *τ*_2_ was found. These patterns were independent of posture.

**Conclusion:**

The results are fully compatible with the tested hypothesis. They provide the first direct demonstration that vagal blockade, while suppressing HR *φ*_1_, did not affect *φ*_1_ of CO.

## Introduction

The oxygen uptake ($\dot{V}$O_2_) kinetics at the onset of a moderate-intensity exercise is characterised by two distinct phases (Whipp et al. 1982), which have been modelled as a sum of two exponentials (Barstow and Molé 1987). The first rapid phase (*φ*_1_) has been ascribed to a sudden increase in cardiac output (CO) and was called “cardiodynamic phase” (Wasserman et al. 1974). The demonstration of a rapid increase in CO at exercise onset is the strongest piece of evidence supporting this interpretation (Cummin et al. 1986; Eriksen et al. 1990; De Cort et al. 1991; Yoshida et al. 1993). The second or primary phase (*φ*_2_), slower than *φ*_1_, was attributed to muscle metabolic adaptations (di Prampero 1981; Whipp and Ward 1990; Poole and Jones 2012; Ferretti 2015). Application of the double exponential model to the analysis of the CO kinetics at exercise onset led to the demonstration that the *φ*_1_ for $\dot{V}$O_2_ kinetics may be entirely explained by the *φ*_1_ for CO (Faisal et al. 2009; Lador et al. 2006, 2008).

Two different mechanisms have been hypothesised to explain the *φ*_1_ for CO. On one side, some authors postulated that a sudden fall in vagal activity occurs at exercise start, leading to a fast increase in heart rate (HR) and, thus, in CO (Fagraeus and Linnarsson 1976; Fontolliet et al. 2018; Lador et al. 2006, 2008): this is the so-called vagal withdrawal concept, although even an incomplete withdrawal of vagal modulation of HR was recently considered sufficient to explain the phenomenon (White and Raven 2014). On the other side, some authors postulated that at exercise start there is a rapid increase in venous return, due to sudden displacement of blood from the contracting muscles to the heart by muscle pump action. This would lead to an immediate increase in stroke volume (SV) and, thus, in CO (Laughlin 1987; Leyk et al. 1994; Linnarsson et al. 1996; Wieling et al. 1996; Sundblad et al. 2000; Schneider et al. 2002; Stenger et al. 2012). Fagoni et al. (2020) argued that these two postulated mechanisms may coexist and are not mutually exclusive, one driven by HR, and the other by SV.

In the present study, the vagal withdrawal concept for CO kinetics, which thus far has only received indirect support, was tested. Fagraeus and Linnarsson (1976) demonstrated the disappearance of *φ*_1_ for HR after complete vagal withdrawal with atropine without measuring CO. Lador et al. (2008) demonstrated that in acute hypoxia (i.e. a condition characterised by lower vagal activity than normoxia) the amplitude of *φ*_1_ (*A*_1_) for HR and CO kinetics was lower than in normoxia. Moreover, a rapid initial component in HR on-kinetics was not found in heart transplant recipients (Grassi et al. 1997).

All this evidence was indirect. The vagal withdrawal concept for CO was not tested under vagal blockade so far. Vagal blockade inhibits the parasympathetic modulation of heart activity already at rest. Therefore, no vagal withdrawal should occur at exercise onset under vagal blockade. If the hypothesis by Fagoni et al. (2020) is correct, we should expect that, under vagal blockade, the *A*_1_ for HR would reduce to nil, whereas the *A*_1_ for CO, though having a smaller size than in control condition, would still be visible and positive, due to the sudden increase in SV.

To test the vagal withdrawal concept, we aimed to determine the $\dot{V}$O_2_, CO, and HR kinetics during vagal blockade with atropine, and to compare these responses with control conditions. We analysed the data using the double exponential model and we computed the characteristic parameters of *φ*_1_ (amplitude and time constant) for the three measured variables.

## Methods

### Subjects

Nine healthy non-smoking men subjects took part in the study. They were (mean ± SD) 23 ± 3 years old, 180 ± 3 cm tall, and 77 ± 6 kg heavy.

### Protocol

Subjects were asked to come to the laboratory on two separate days, one for the protocol under vagal blockade and one for the control condition. During each testing session, the protocol was carried out in both upright and supine position, administered in a random order. Parasympathetic blockade was obtained by injecting a single dose of 0.04 mg/kg (mean 3.06 ± 0.23 mg, range 2.7–3.4 mg) of atropine, using a short indwelling venous catheter in an antecubital vein (Yasue et al. 1986; Morikami et al. 1988; Goldberger et al. 2001; Ferretti et al. 2005; Fontolliet et al. 2018).

After participants' preparation and instrument calibration, 3-min rest monitoring recordings were performed, during which blood sampling and CO determination with the acetylene method were done. Then, the subject was asked to perform three exercise transients from 0 to 80 W. The starting signal was provided by a verbal countdown. The flywheel was not pre-accelerated. The first bout lasted 6 to 7 min, to allow blood sampling and CO determination with the acetylene method after 5 min of exercise (at exercise steady state), while the second and third bouts lasted 5 min, at the end of which only blood sampling was taken. Each exercise bout was followed by 6 min of recovery, during which [La]_b_ blood sampling was performed at minutes 1, 3, and 5.

### Measurements

The time course of oxygen and carbon dioxide partial pressures throughout the respiratory cycles were continuously monitored by a mass spectrometer (Balzers Prisma, Balzers, Liechtenstein) calibrated against gas mixtures of known composition. The inspiratory and expiratory ventilation was measured by an ultrasonic flowmeter (Spiroson^®^, ECO MEDICS AG, Duernten, Switzerland) calibrated with a 3 l syringe. HR was continuously measured by electrocardiography (Elmed ETM 2000, Heiligenhaus, Germany). Continuous recordings of arterial pulse pressure were obtained at a fingertip of the left arm by means of a non-invasive cuff pressure recorder (Portapres^®^, Finapres^®^ Medical Systems, Enschede, The Netherlands). Steady-state CO values were obtained by means of the open circuit acetylene method (Barker et al. 1999), with a procedure previously described (Lador et al. 2006), implying determination of partition coefficients for acetylene (Meyer and Scheid 1980). The rationale of this methodological approach was previously discussed (Lador et al. 2006, 2008).

All signals were digitalized in parallel by a 16-channel A/D converter (MP150 system with AcqKnowledge acquisition and analysis software, BIOPAC^®^ Systems Inc., Goleta, CA, USA) and stored on a computer. The acquisition rate was 400 Hz.

Blood lactate concentration ([La]_b_) was measured by an electro enzymatic method (Eppendorf EBIO 6666, Erlangen, Germany) on 10 μL blood samples taken from the right earlobe. Capillary blood gas composition and pH were measured by microelectrodes (Instrumentation Laboratory Synthesis 10, Lexington, MA, USA) on 50 μL blood samples taken from the right earlobe.

### Ergometers

In an upright position, subjects exercised on a standard electrically braked cycle ergometer (Ergo-metrics 800S, Ergo-line, Bitz, Germany). In the supine position, an electrically braked arm-cycle ergometer (Ergoselect 400, Ergoline GmbH, Bitz, Germany), modified for leg pedalling in supine posture, was used. Subjects wore race cycling shoes, allowing fixation of their feet to the pedals. The subjects were asked to keep a pedalling frequency between 60 and 80 rpm (visual feedback). The pedalling frequency was recorded, and its sudden increase at the exercise onset and decrease at the exercise offset were used as markers to precisely identify the start and the end of exercise. In both positions, the electro-mechanical characteristics of the ergometers were such as to permit workload application in less than 50 ms.

### Data treatment

Oxygen and carbon dioxide partial pressures traces were aligned with the flowmeter traces and breath-by-breath $\dot{V}$O_2_ and carbon dioxide output ($\dot{V}$CO_2_) were then computed off-line by means of a modified version of Grønlund’s algorithm (1984) run in LabVIEW^®^ environment (LabVIEW^®^ 5.0, National Instruments™, Austin, TX, USA). The characteristics and the physiological implications of Grønlund’s algorithm have been previously discussed elsewhere (Capelli et al. 2001, 2011; Lador et al. 2006).

Arterial blood pressure profiles were analysed to obtain beat-by-beat values using the Beatscope^®^ software (Finapres^®^ Medical Systems, Enschede, The Netherlands). The same software also provided SV by means of the Modelflow method (Wesseling et al. 1993). Beat-by-beat CO was computed as the product of single-beat SV times the corresponding single-beat HR. The data were then corrected for method’s inaccuracy, as previously described (Kenfack et al. 2004; Tam et al. 2004; Lador et al. 2006). Individual correction factors were calculated at steady state, using the open-circuit acetylene CO values as reference, and applied during dynamic states with rapid changes in CO (van Lieshout et al. 2003). The calibration factors were the same at rest (1.02 ± 0.29) and exercise (1.03 ± 0.24, non-significant).

The three transitions of either $\dot{V}$O_2_, CO or HR were time aligned, by setting the time of exercise start as time zero for the analysis of the on-kinetics. Then CO, HR and $\dot{V}$O_2_ traces from the three repetitions were pooled together for each subject without interpolation (Bringard et al. 2014). For each variable, the time course upon exercise onset was analysed by the double exponential model (Barstow and Molé 1987; Lador et al. 2006):

1 $$f\left(t\right)=b+{A}_{1}\theta \left(t-{d}_{1}\right)\left(1-{e}^{-\frac{t-{d}_{1}}{{\tau }_{1}}}\right)+{A}_{2}\theta \left(t-{d}_{2}\right)\left(1-{e}^{-\frac{t-{d}_{2}}{{\tau }_{2}}}\right),$$

where *b*, *A*, *d*, and *τ* are the baseline values at rest, the amplitude, the time delay, and the time constant, respectively. The subscripts 1 and 2 refer to the *φ*_1_ and the *φ*_2_ of the on-kinetics, respectively. ϴ is the Heaviside function (ϴ(*t*) = 0 if *t* < 0 and ϴ(*t*) = 1 if *t* ≥ 0). When *A*_1_ = 0, the second term of the right-hand branch of Eq. (1) cancels out. In this case, (i) *τ*_1_ becomes meaningless and cannot be computed so that for *τ*_1_ “*n*” may be less than the number of subjects, and (ii) Eq. (1) reduces to a single exponential equation, with one time constant, equal to *τ*_2_, and overall amplitude of response equal to *A*_2_. If the biexponential model provided *A*_1_ not significantly different from zero, *A*_1_ was set as equal to 0 L min^−1^, *τ*_1_ was neglected as meaningless, and a mono-exponential analysis of *φ*_2_ was applied.

The total sum of the squared 2 residuals (RESNORM variable) was also systematically calculated by MATLAB function. The comparison of the RESNORM between double exponential and mono-exponential analysis was used as a quality check of the model. In fact, whenever we neglected *τ*_1_ and fitted only *φ*_2_, the RESNORM analysis demonstrated that a double exponential fit did not improve the quality of fitting (RESNORM of mono-exponential fitting less than 1% lower than with double exponential fitting whenever *A*_1_ = 0 L min^−1^).

Steady state means were calculated as the average values of the last min of rest and of the 5th min of exercise. Arterial-venous O_2_ concentration difference (CaO_2_–C$\overline{v }$O_2_) was calculated at rest and at exercise steady state as the ratio between $\dot{V}$O_2_ and CO (Adami et al. 2014; Ferretti et al. 2017).

### Statistics

Data are given as mean and standard deviation (SD) of the values obtained for each parameter from the average superimposed files of each subject, to account for inter-individual variability. Difference between rest and exercise steady state was computed as the latter minus the former (Δ). The atropine-induced effects on the primary outcomes were analysed by a 2-way ANOVA (drug and X body position). When applicable, a Tukey post hoc test was used to locate significant differences. The results were considered significant if *p* < 0.05. The parameters of the models were estimated with a weighted non-linear least squares procedure (Bringard et al. 2014; Carson et al. 1983), implemented in MATLAB (version 7.9.0, MathWorks^®^, Natick, MA, USA).

## Results

### Resting and exercise steady-state phase

The mean values of measured and calculated variables at rest and during exercise steady state for all conditions are reported in Table 1. HR in atropine, whether supine or upright, was higher than the control condition, both at rest (*p* < 0.05) and at exercise (*p* < 0.05). At rest in the supine position and during exercise in both postures, SV was lower under atropine than in control (*p* < 0.05). In upright posture, SV was always lower than in supine posture (*p* < 0.001), except in control during exercise (*p* = 0.41). Resting CO was higher in atropine than in control, whether supine or upright (*p* < 0.05). In atropine, CO was lower in the upright than in supine posture, both at rest and during exercise (*p* < 0.05). Oxygen uptake value did not change at rest (*p* > 0.18). During exercise under vagal blockade, $\dot{V}$O_2_ was lower in upright than in supine posture (*p* = 0.01). A similar non-significant trend occurred also in control (*p* > 0.99).

**Table 1:** _2_  Mean values of measured and calculated variables at rest and during exercise steady state, in control condition and with atropineMean values of measured and calculated variables at rest and during exercise steady state, in control condition and with atropine

			Control		Atropine	
			Supine	Upright	Supine	Upright
HR	Rest	min^−1^	61 ± 10	71 ± 8	105 ± 9^#^	109 ± 9^#^
	Exercise	min^−1^	110 ± 10	105 ± 10	139 ± 13^#^	129 ± 13^#^
SV	Rest	mL	97 ± 13	79 ± 13*	78 ± 10^#^	66 ± 9*
	Exercise	mL	120 ± 15	108 ± 9	103 ± 16^#^	83 ± 11^#,^*
CO	Rest	L min^−1^	5.9 ± 0.9	5.6 ± 1.0	8.2 ± 0.6^#^	7.1 ± 0.6^#,^*
	Exercise	L min^−1^	12.9 ± 1.8	11.2 ± 1.3	14.1 ± 2.3	10.6 ± 1.6*
$\dot{V}$O_2_	Rest	L min^−1^	0.44 ± 0.06	0.51 ± 0.09	0.49 ± 0.08	0.49 ± 0.08
	Exercise	L min^−1^	1.59 ± 0.10	1.53 ± 0.25	1.67 ± 0.09	1.43 ± 0.13*

Mean values of measured and calculated variables at rest and during exercise steady state, in control condition and with atropine

*HR* heart rate, *SV* stroke volume, *CO* cardiac output, $\dot{V}$*O*_*2*_ oxygen consumption, *min*^*−1*^ beat per minute

*p < 0.05 vs. supine position, within the same condition

^#^*p* < 0.05 vs. control, within the same posture

The values for [La]_b_, pH, and partial pressure of CO_2_ (PCO_2_) in capillary blood are reported in Table 2. We observed no differences between rest and exercise (respectively, *p* = 0.67, 0.21 and 0.07) or between atropine and control (respectively, *p* = 0.12, 0.94 and 0.08) in any of the investigated conditions.

**Table 2:** Mean values of [La]b pH and PCO2 at rest and during exercise steady state, in control condition and with atropine

				Control		Atropine	
				Supine	Upright	Supine	Upright
[La]_b_		Rest	L min^−1^	0.96 ± 0.12	0.63 ± 0.05	1.13 ± 0.12	0.71 ± 0.05
		Exercise	L min^−1^	1.00 ± 0.09	0.68 ± 0.10	1.20 ± 0.01	0.68 ± 0.06
pH		Rest		7.44 ± 0.00	7.44 ± 0.00	7.44 ± 0.00	7.44 ± 0.00
		Exercise		7.43 ± 0.00	7.43 ± 0.00	7.43 ± 0.01	7.43 ± 0.00
PCO_2_		Rest	mmHg	37.0 ± 0.6	36.1 ± 0.3	37.3 ± 0.5	38.0 ± 0.6
		Exercise	mmHg	38.7 ± 0.9	36.5 ± 0.4	38.9 ± 0.6	38.7 ± 0.3

Mean values of [La]b pH and PCO_2_ at rest and during exercise steady state, in control condition and with atropine

*[La]*_*b*_ blood lactate concentration, *pH* hydrogen potential, *PCO*_*2*_ partial pressure of CO_2_

**Fig. 1:**
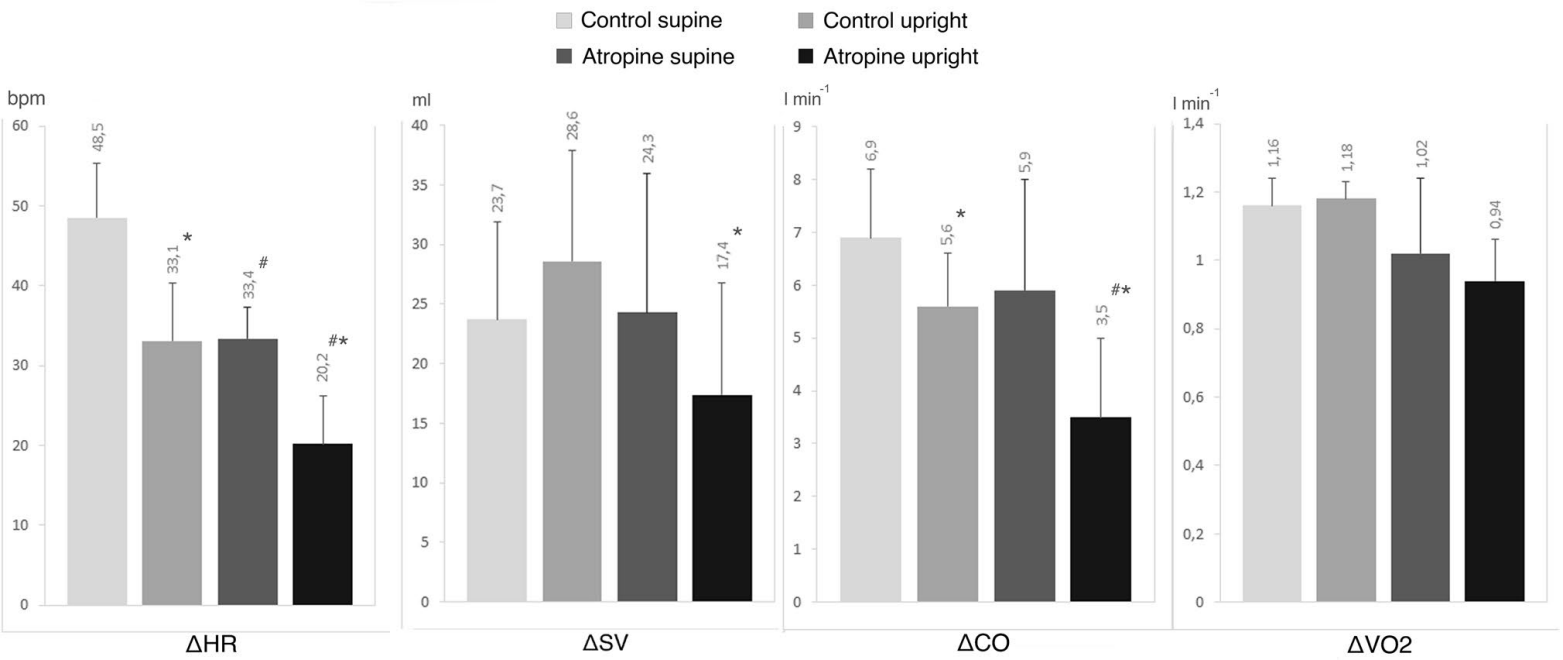
Difference (Δ) in heart rate, cardiac output, stroke volume and oxygen consumption between rest and exercise steady state in supine and upright position and for control situation and with atropine (*n* = 9). $\dot{V}$*O*_*2*_ oxygen consumption, *CO* cardiac output, *HR* heart rate, *SV* stroke volume, *p < 0.05 vs. supine position; ^#^*p* < 0.05 vs. control

Figure 1 reports the difference (Δ) between the exercise steady state and the resting value for the investigated parameters. This difference is indicative of the overall amplitude of response during the rest–exercise transition for each parameter. The ΔHR was smaller under atropine than in control, when exercise was performed in both supine and upright position (*p* < 0.05). This was not the case for SV (*p* = 0.85 and 0.06) and $\dot{V}$O_2_ (*p* = 0.98 and 0.58) therefore, ΔCO only tended to decrease in atropine with respect to control (*p* = 0.20 and 0.005).

**Fig. 2:**
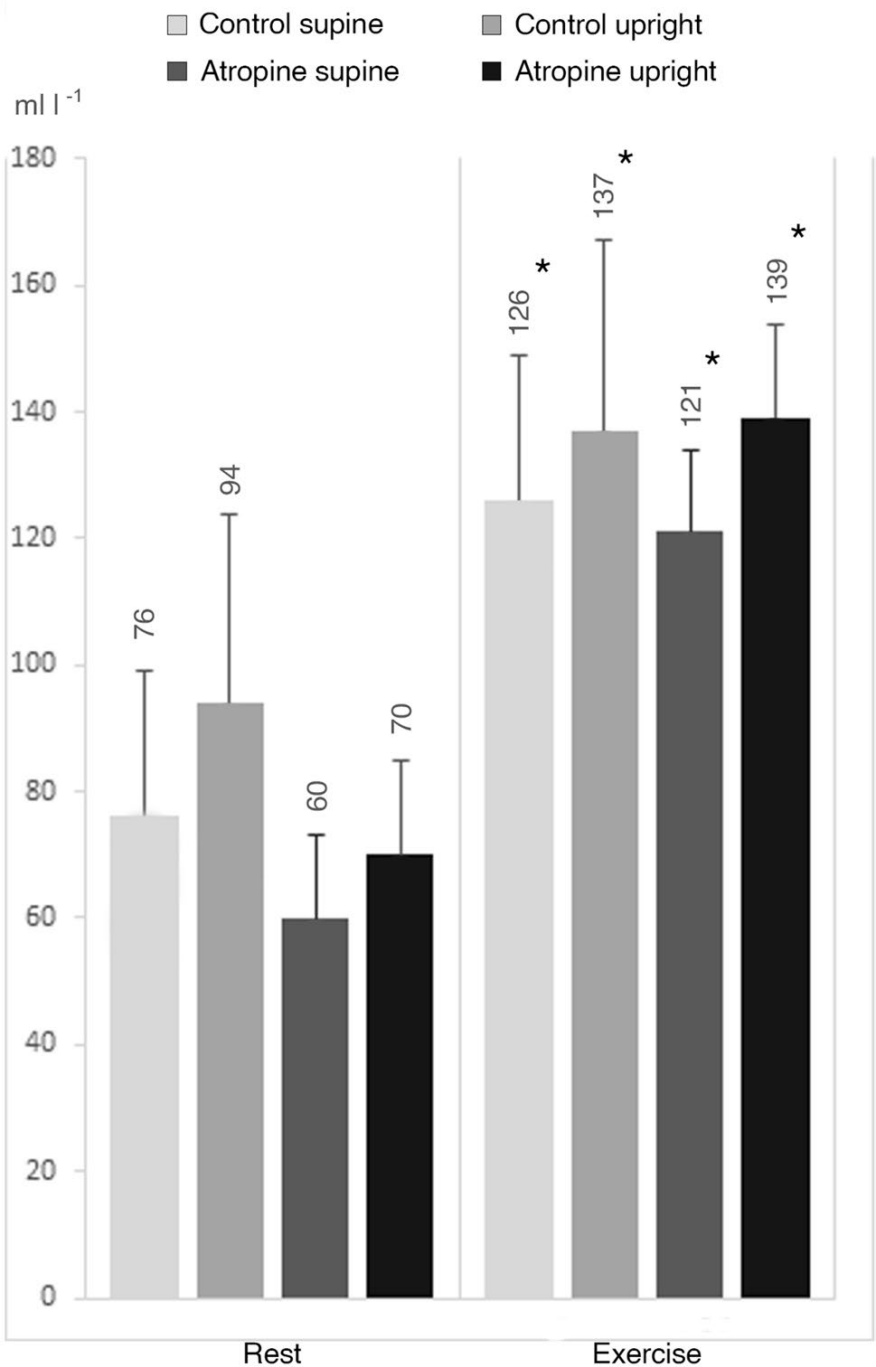
Calculated arterial-venous O_2_ difference between rest and exercise steady state in supine and upright position and for control situation and with atropine. *CaO*_*2*_*–C*$\overline{v }$*O*_*2*_ arterial-venous O_2_ difference; *p < 0.05 vs. rest

Calculated CaO_2_–C$\overline{v }$O_2_ values are reported in Fig. 2. No differences were found among all four conditions whether at rest or at exercise steady state. As expected, CaO_2_–C$\overline{v }$O_2_ was higher at exercise than at rest in all conditions (*p* < 0.05).

### Kinetics responses

**Fig. 3:**
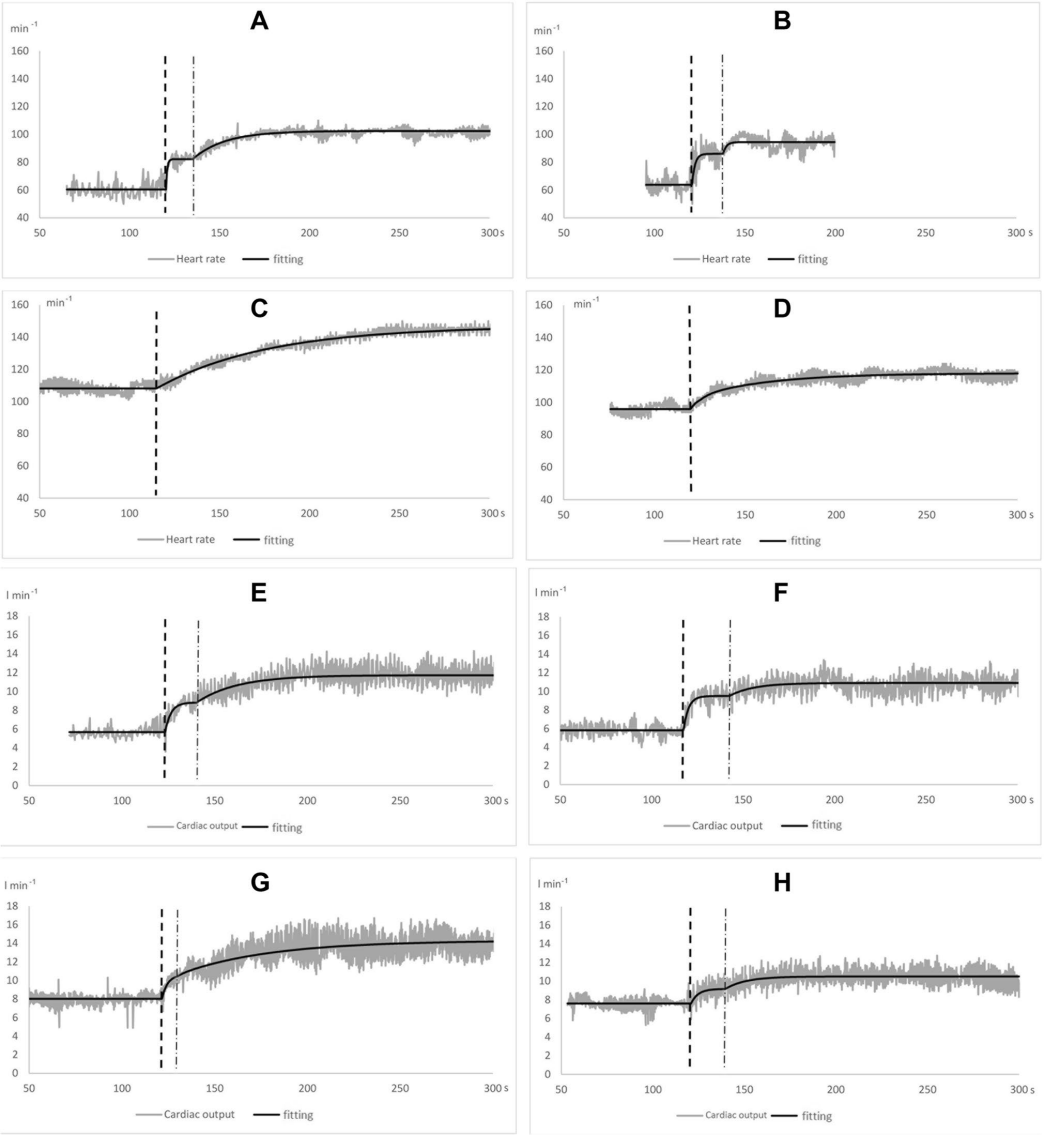
Individual examples of the time course of heart rate and cardiac output in supine and upright position and for control situation and with atropine. Single beat data (grey trace) and corresponding fitting function (black line) are reported. **A**–**D** heart rate (min^−1^); **E**–**H**: cardiac output (L min^−1^). Conditions: **A**, **E** control supine; **B**, **F** control upright; **C**, **G** atropine supine; **D**, **H** atropine upright. All traces were fitted by the double exponential model (sum of two exponentials). However, fitting of the traces reported in **C** (atropine supine, heart rate) and **D** (atropine upright, heart rate) yielded phase I amplitude equal to zero: thus, they were fitted as monoexponential functions, due to the absence of phase I, as detailed in the method section

Individual examples of the time course of HR and CO kinetics upon exercise onset are shown in Fig. 3.

**Fig. 4:**
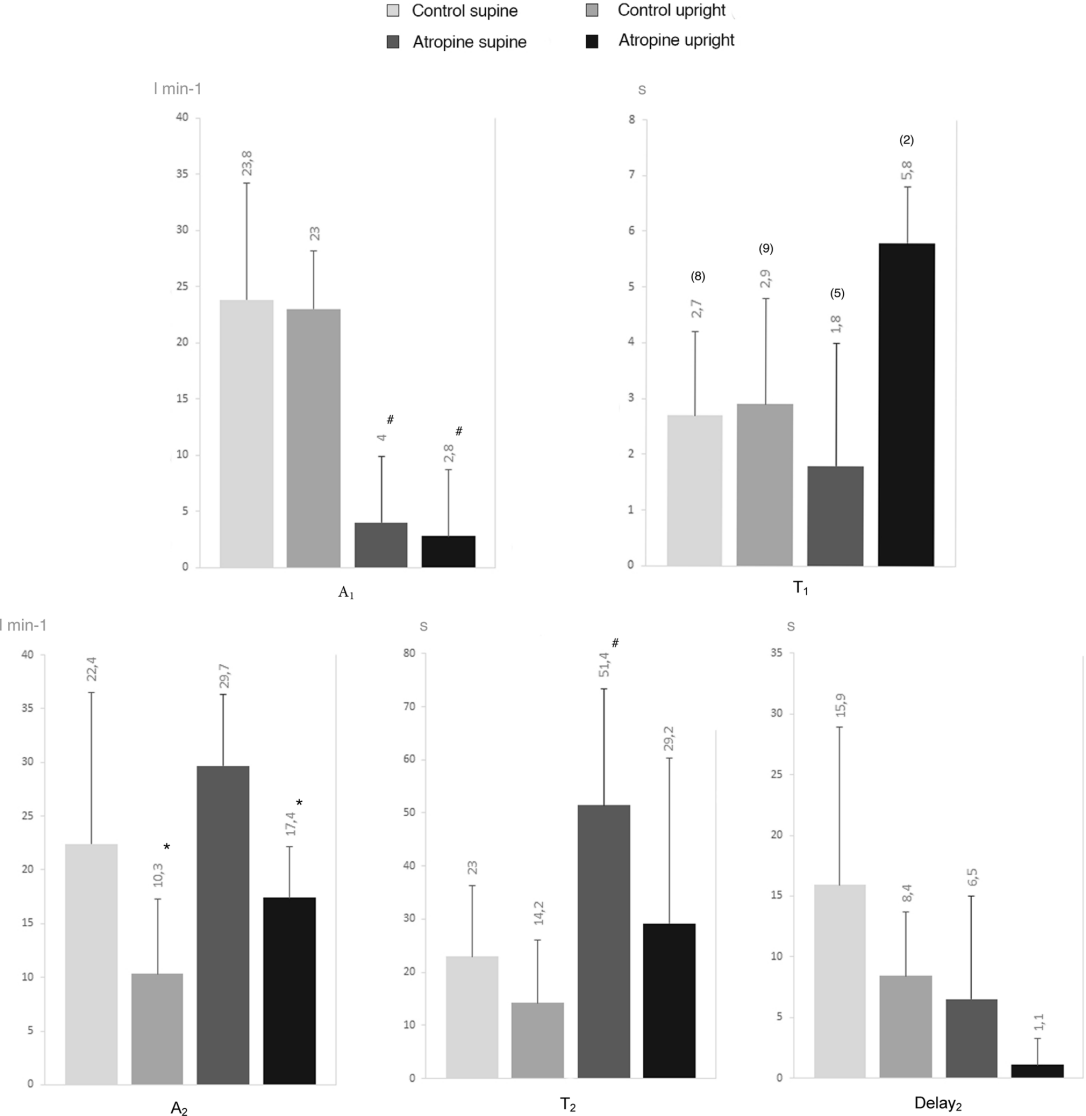
Heart rate kinetics between rest and exercise steady state in supine and upright position and for control situation and with atropine. *HR* heart rate, *A* amplitude, *τ* time constant. The subscripts 1 and 2 refer to the *φ*_1_ and the *φ*_2_ of the bi-exponential model; (*n*): number of subjects on which *τ*_1_ was computed; *p < 0.05 vs. supine position; ^#^*p* < 0.05 vs. control

In no case ${d}_{1}$ differed from 0, so we retained no time delay for *φ*_1_. Parameters of the double exponential equation describing HR kinetics are shown in Fig. 4. In control, *φ*_1_ was visible in the whole cohort in upright and supine position, except for one subject in the latter body position. Under atropine, *φ*_1_ was identified only in five subjects in supine and in two subjects in upright position. The magnitude of *A*_1_ was smaller in atropine than in control (*p* < 0.001 and 0.05). Similar results were found when *A*_1_ was expressed as a percentage of ΔHR (in the supine position, *A*_1_ was 50 ± 21% in control and 11 ± 14% in atropine, *p* < 0.05; in the upright position, *A*_1_ was 70 ± 16% in control and 11 ± 22% in atropine, *p* < 0.05). *A*_2_ was smaller in upright position both in control (*p* < 0.05) and under vagal blockade (*p* < 0.001). Concerning *τ*_1_, the lack of values under atropine, especially in an upright posture (*n* = 2) makes a statistical comparison meaningless. In supine posture, *τ*_2_ was larger in atropine than in control (*p* < 0.05).

**Fig. 5:**
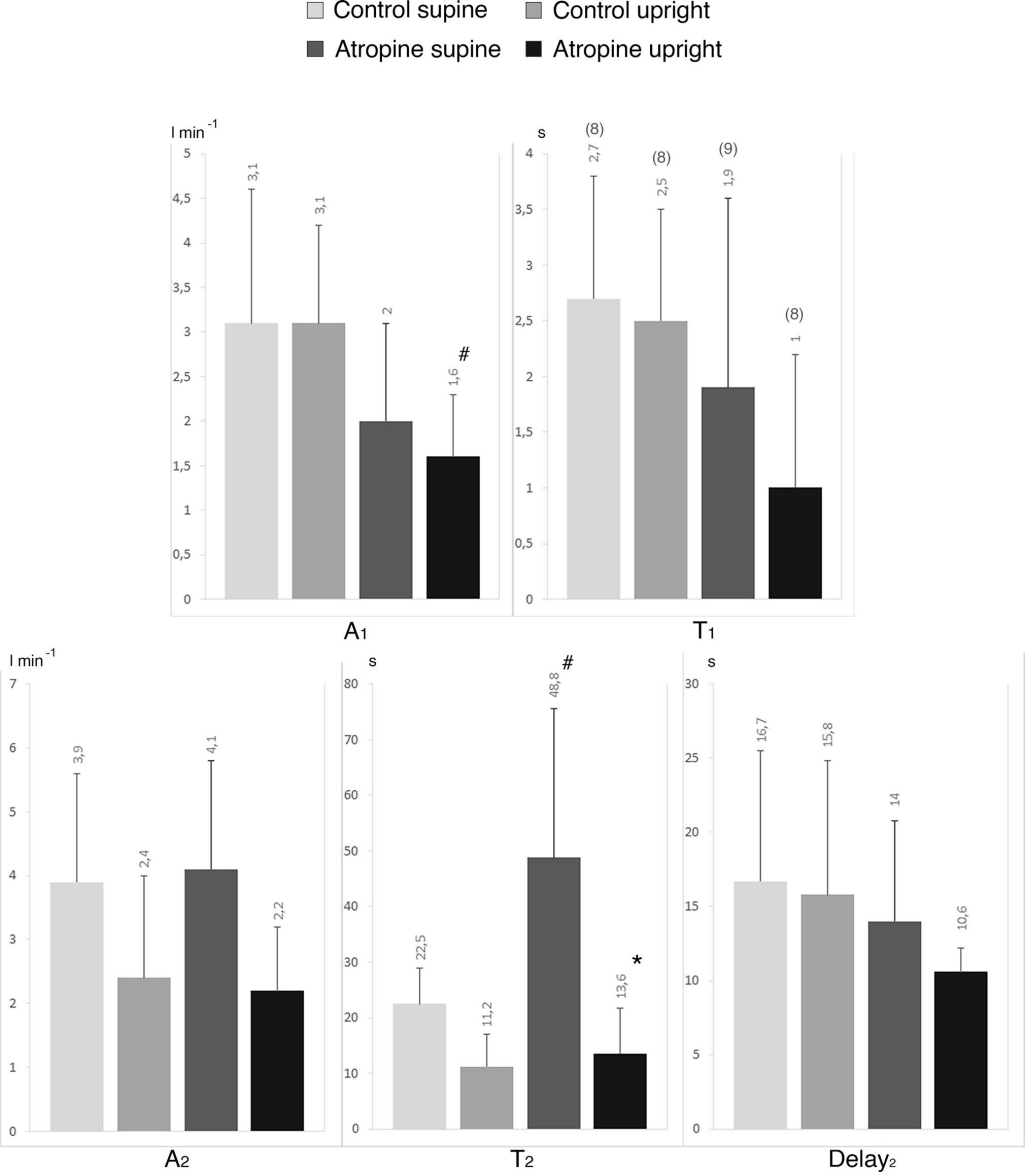
Cardiac output kinetics between rest and exercise steady state in supine and upright position and for control situation and with atropine. *CO* cardiac output, *A* amplitude, *τ* time constant. The subscripts 1 and 2 refer to the *φ*_1_ and the *φ*_2_ of the bi-exponential model; (*n*): number of subjects on which *τ*_1_ was computed. *p < 0.05 vs. supine position; ^#^*p* < 0.05 vs. control

The parameters of the double exponential equation describing the CO kinetics at the exercise onset are shown in Fig. 5. For CO, *A*_1_ was lower in atropine than in control in the upright position (*p* < 0.05). No significant difference in *τ*_1_ was found, neither for body position (*p* = 0.98 and 0.51) nor for atropine (*p* = 0.57 and 0.09). The values of *A*_2_ were the same in all conditions (*p* = 0.97 and 0.38). In atropine, *τ*_2_ was lower in the upright than supine position (*p* < 0.001). In supine posture, *τ*_2_ was higher in atropine than in control (*p* < 0.05). Notwithstanding, we also remark that the *τ*_2_ mean value in atropine was affected by an outlier value (*τ*_2_ of 101.4, at the limit of two SD above the group mean).

**Fig.6:**
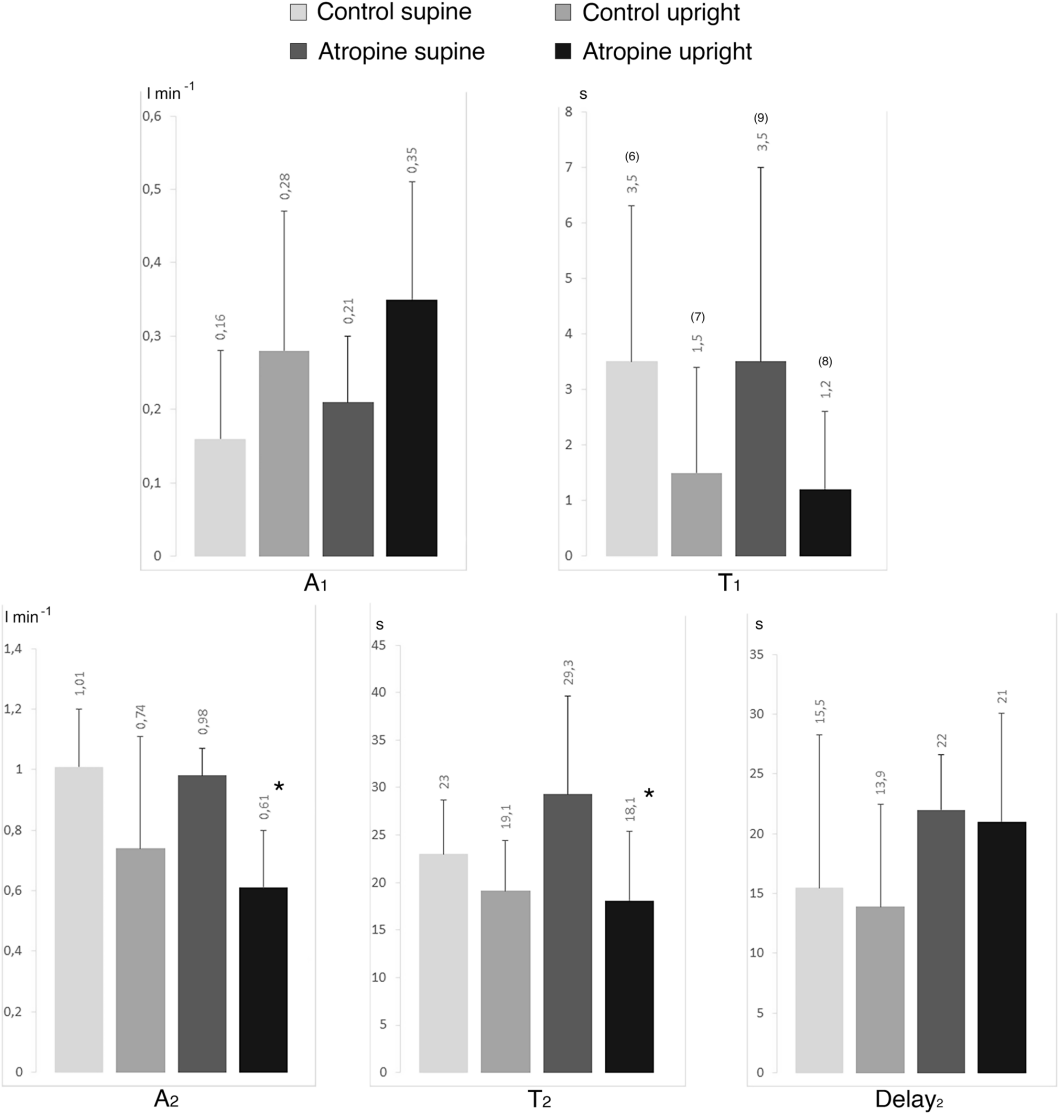
Oxygen consumption kinetics at rest and exercise steady state in supine and upright position and for control situation and with atropine. $\dot{V}$*O*_*2*_ oxygen uptake, *A* amplitude, *τ*: time constant. The subscripts 1 and 2 refer to the *φ*_1_ and the *φ*_2_ of the bi-exponential model; (*n*): number of subjects on which *τ*_1_ was computed. *p < 0.05 vs. supine position

Double exponential analysis was applied to $\dot{V}$O_2_ kinetics in all subjects, in all experimental conditions. Parameters of the double exponential equation describing $\dot{V}$O_2_ kinetics at the exercise onset are shown in Fig. 6. In atropine, *A*_2_ and *τ*_2_ were lower in the upright than in supine position (*p* < 0.05).

## Discussion

This study provides the first demonstration of the effects of parasympathetic blockade on the cardiorespiratory response during exercise transients. Under atropine, *φ*_1_ for HR was almost completely suppressed suggesting that the *A*_1_ for CO response kinetics was determined uniquely by the changes in SV. The lower values of *A*_1_ for CO found during exercise performed after atropine injection, compared to the control condition, corresponds to the contribution of HR to the *φ*_1_ in CO kinetics. These findings are in agreement with what we originally hypothesized and they represent the most current quantitative demonstration of the role of the inhibition of vagal modulation of HR at exercise start in determining the early CO response. The pattern followed by the different pertinent variables are analysed herewith, as an introduction to a holistic discussion of *φ*_1_ kinetics.

### Heart rate

The observation that under vagal blockade in both postures the *A*_1_ for HR was largely reduced, if not altogether suppressed, was not surprising. Fagraeus and Linnarsson (1976) were the first to analyse the rapid response of HR at exercise onset on a beat-by-beat basis. Although they did not model the transient kinetics, they observed that atropine remarkably slowed the overall HR response at exercise onset. Craig and Cummings (1963) reported what they called a “blocking effect” of atropine on the initial rapid HR increase, in a study in which HR was averaged over 10 s periods, thereby obtaining a dampened response. Later, using the supine knee extension model, Toska et al. (1994) confirmed that the initial rapid increase of HR at exercise onset was altered under atropine injection, thus demonstrating that the effect was independent of posture. Their results are in line with the present ones, as long as we found no differences concerning *A*_1_ for HR between the two investigated postures. Lador et al. (2006) were the first who applied a double exponential model to the analysis of the HR kinetics, by extending the application range of the model developed by Barstow and Molé (1987) for the study of $\dot{V}$O_2_ kinetics. Based on the results of Fagraeus and Linnarsson (1976), Lador et al. (2006) postulated that the *A*_1_ for HR was the result of withdrawal of vagal tone at exercise start. The same group provided two further indirect pieces of evidence supporting that conclusion. First, they found that the *A*_1_ for HR was smaller in acute hypoxia than in normoxia (Lador et al. 2008) since in hypoxia, resting modulation of HR by the vagal system is blunted with respect to normoxia (Xie et al. 2001), resting HR is higher and *A*_1_ for HR is lower in the former than in the latter case. Second, if vagal withdrawal occurs, its occurrence is immediate at the exercise start (Bringard et al. 2017).

As expected (Nyberg 1981; Toska and Eriksen 1993; Wray et al. 2001), the resting HR of our study was higher in atropine than in control (Table 1), whereas the *A*_1_ was lower (Fig. 3). Therefore, the vagal modulation of the heart was already suppressed at rest, and no vagal withdrawal was possible at exercise onset. In control, the sum of resting HR (Table 1), and the *A*_1_ of HR provides the theoretical *φ*_1_ asymptote for HR. In control, this sum resulted 85 and 94 min^−1^ supine and upright, respectively. This means that in *φ*_1_ the HR has increased by 24 min^−1^ supine and by 23 min^−1^ upright above the respective resting values. Under atropine, the *φ*_1_ asymptote for HR was 109 and 111 min^−1^, supine and upright, respectively. The former value is identical to the resting HR supine, the latter was only 5 min^−1^ higher than the resting HR value upright.

In heart transplant recipients, the basal HR at rest is in the range 100–112 min^−1^ (Pflugfelder et al. 1987; Convertino et al. 1990; Shephard 1992; Backman et al. 1997; Grassi et al. 1997; Strobel et al. 1999; Ferretti et al. 2002; Kaufmann et al. 2007). In atropine, independently of the posture, the resting HR values are fully within this range, indicating complete suppression of cardiac vagal control by atropine. Coherently, the *φ*_1_ asymptote for HR under atropine remained practically equal to the resting HR. This is due to the extremely low *A*_1_ amplitude in atropine, as a result of a lack of vagal withdrawal at exercise start (the vagus is already inhibited at rest). Our hypothesis was that vagal withdrawal explains the HR increase in *φ*_1_ at exercise onset, and these findings confirmed our original assumption. Nevertheless, the *φ*_1_ asymptote for HR was higher under atropine than in control. If our hypothesis was correct, these data would suggest that in control, at the power used for this study, the withdrawal of vagal tone upon exercise start was incomplete, as the HR at the asymptote of *φ*_1_ remains lower than that we would expect under parasympathetic blockade. The equal magnitude of *A*_1_ in supine and upright indicates that the HR response in *φ*_1_ is independent of body posture.

In *φ*_2_, the time constant was similar to that of previous studies (Lador et al. 2006, 2013). *A*_2_ is smaller in the upright than supine in control, a condition in which both branches of the autonomic nervous system are active. Under atropine, the resting HR is the same, yet the *A*_2_ is smaller upright than supine. This may mean that resting HR is mostly determined by the parasympathetic heart modulation, whereas the degree of sympathetic system stimulation during exercise determines the amplitude of the *φ*_2_ HR response. This interpretation would be compatible with the hypothesis proposed by Lador et al. (2006) of a predominant sympathetic role in *A*_2_ for HR.

The sum of *A*_1_ plus *A*_2_ is the overall HR response to exercise and it should correspond to ΔHR. A comparison between these two parameters shows that this is the case indeed. In fact, the sum of *A*_1_ plus *A*_2_, expressed in min^−1^, was 46 ± 5 in control supine, 34 ± 7 in control upright, 33 ± 4 in atropine supine, 20 ± 6 in atropine upright. A comparison of these values with the ΔHR data reported in Fig. 1 shows that the former corresponds well to the latter in each condition.

### Cardiac output

Different patterns appear concerning CO responses. Contrary to HR, CO shows a positive *φ*_1_ response under atropine and control experimental conditions. However, *A*_1_ is smaller in atropine than in control, because in atropine the *φ*_1_ response is entirely due to SV response, considering the *A*_1_ for HR negligible. In this condition, it is possible to distinguish the SV role from the HR role in determining the *φ*_1_ CO response. Both in supine and in upright posture, the CO response due to SV in control is equal to the *A*_1_ for CO in atropine; the fraction of the CO response due to HR in control is equal to the difference between the *A*_1_ for CO in control and the *A*_1_ for CO in atropine. Is this difference compatible with the *φ*_1_ increase for HR? This is hard to establish because SV is also a matter of telediastolic volume, which depends on the diastole duration, which is shorter in atropine than in control. Nevertheless, it is a matter of fact that these results show that atropine reduced *A*_1_ for CO but did not suppress it. It was reduced because of the suppression of *A*_1_ for HR. Thus, the remaining yet lower CO response in *φ*_1_ under atropine cannot be a consequence of vagal withdrawal, because there is no vagal withdrawal between rest and exercise in this case: the parasympathetic system is inhibited all time long. It cannot be due to increased sympathetic stimulation at exercise, because the *φ*_1_ duration is too short for that. If sympathetic stimulation plays a role, it would be responsible only for the *φ*_2_ CO response, both for its chronotropic and inotropic effect on the heart.

These results confirm the hypothesis of the muscle pump mechanism, originally proposed by Sheriff et al. (1993) on animals and later resumed to explain the CO increase in heart transplant recipients (Meyer et al. 1998). This mechanism, which was finally demonstrated also in healthy humans under Lower Body Negative Pressure (Fagoni et al. 2020), may well operate to determine the entire *φ*_1_ CO response when the vagal system is blocked and therefore there is no HR contribution to the *φ*_1_ CO kinetics.

### Oxygen uptake

Concerning the $\dot{V}$O_2_ kinetics, *φ*_1_ was visible in all conditions. There was no decrease of following atropine injection. Lador et al. (2006) showed, with the Fick principle, that the *φ*_1_ amplitude of the CO kinetics explains entirely the *φ*_1_ amplitude of the $\dot{V}$O_2_ kinetics, in line with the cardiodynamic hypothesis of Wasserman et al. (1974). Because of a delay between muscle O_2_ consumption and lung O_2_ uptake (DeLorey et al. 2003), we can assume that the composition of *A*_1_ mixed venous blood remains unchanged during the first seconds of exercise, and thus arterial-venous O_2_ difference (CaO_2_–C$\overline{v }$O_2_) stays equal to that at rest. In fact this assumption is not in clear contrast with the kinetics of CaO_2_–C$\overline{v }$O_2_ that we could estimate after Casaburi et al (1989), who determined a kinetics of mixed venous oxygen saturation (S$\overline{v }$O_2_) with a time resolution of 4 s and characterised by a half-time of 32 s (time constant of approximately 46 s): within the time resolution of *φ*_1_ this would imply a negligible error indeed. Thus, if we assume that CaO_2_–C$\overline{v }$O_2_ stays equal to that at rest, and we accept the cardiodynamic hypothesis of Wasserman et al (1974), we can thus predict the change in *A*_1_ for $\dot{V}$O_2_ by applying the Fick principle and multiplying *A*_1_ for CO and CaO_2_–C$\overline{v }$O_2_ at rest (Lador et al. 2006). The predicted *A*_1_ for $\dot{V}$O_2_ is compared with the one that we determined in this study using the double exponential model in Table 3.

**Table 3:** Measured and recalculated $\dot{V}$O_2_ kinetics increase at rest and during exercise steady state, in control condition and with atropine

Δ $\dot{V}$O_2_		Control supine *N* = 6	Control upright *N* = 7	Atropine supine *N* = 9	Atropine upright *N* = 8
Measured	L min^−1^	0.23	0.37	0.21	0.39
Recalculated	L min^−1^	0.24	0.33	0.11*	0.11*

Measured and recalculated $\dot{V}$O_2_ kinetics increase at rest and during exercise steady state, in control condition and with atropine

$$\dot{V}$$*O*_*2*_ oxygen uptake

*p < 0.01 vs. measured

The correspondence between recalculated and measured *A*_1_ values for $\dot{V}$O_2_ is excellent in control, as was the case in Lador et al (2006). Conversely, in atropine, the calculated *A*_1_ was much less than the observed *A*_1_ for $\dot{V}$O_2_ (*p* < 0.01 both supine and upright). Several hypotheses have been formulated to explain the CO-induced $\dot{V}$O_2_ increase (De Cort et al. 1991; Yoshida et al. 1993; Leyk et al. 1995; Lador et al. 2006). We highlight an intriguing speculative hypothesis, among these. In a previous study published by our group, the combined analysis of the HR and the mean arterial pressure (MAP) responses, at the onset of exercise, has shown that the *φ*_1_ HR increase, which we attribute to vagal withdrawal, corrects the immediate fall of MAP due to sudden muscle vasodilatation (Bringard et al. 2017). In that study, the increase in HR occurs along a baroreflex sequence, the sensitivity of which is equal to that observed not only at exercise steady state (Iellamo et al. 1994, 1997; Vallais et al. 2009), but also after vagal blockade (Fontolliet et al. 2018). Vagal withdrawal is immediate, then HR follows with some inertia (positive *τ*_1_) along a baroreflex curve whose operational point is displaced from the centring point. We speculate that this baroreflex response may play a role in the coupling of the CO and the $\dot{V}$O_2_ response. If this is correct, then in atropine there would not be the HR increase, but baroreceptor stimulation would persist, thus maintaining an intact ventilatory response, which is not under vagal blockade, contrary to the heart. An analysis of the dynamics of baroreflex responses in the early phase of exercise should help in better focusing and analysing this issue: this should be the object of a further study.

If the baroreflexes are involved in coupling the CO and the $\dot{V}$O_2_ response, the mechanism that determines $\dot{V}$O_2_ *A*_1_: (1) is neural; (2) is generated outside the lungs and is likely to operate at the brain stem level (several hypotheses exist on this subject, see e.g. Lador et al. (2013); (3) has effects on the heart that are mediated by the vagal system; (4) generate an integrated response such that a stronger stimulus is sent to the lungs than to the heart; (5) is calibrated in such a way as to provide $\dot{V}$O_2_ responses that are compatible, in terms of gas exchange, with the CO responses.

It goes without saying that the *φ*_1_ $\dot{V}$O_2_ kinetics, especially if it has a “cardiodynamic” origin, is unrelated to events occurring within the muscles. In fact, the double exponential model, dissociates, at least as far as *φ*_1_ is concerned, from the muscle oxygen consumption kinetics, the control of which is metabolic (Ferretti 2015).

### Steady-state phase

Ekblom et al. (1972) and Fagraeus and Linnarsson (1976) reported that HR was higher at rest, but lower at exercise steady state in atropine compared to control. This result was confirmed later by Toska et al. (1994) and Fisher et al. (2013), the latter using glycopyrrolate to induce parasympathetic blockade. Our results are in line with those of Kelbaek et al. (1991), who reported higher HR and CO, at rest in both upright and supine posture, with atropine than in control. Using a slightly lower intensity of exercise than ours, Kelbaek et al. (1991) reported higher HR and CO at exercise steady-state in an upright posture with atropine, contrary to the present study, where the higher HR was associated with unchanged CO under atropine compared to control. This discrepancy could arise from the difference in exercise intensity used between these studies or from the semi-recumbent posture used by those authors. Ekblom et al. (1972) reported no alteration of CO neither at rest (contrary to the present study) nor during submaximal exercise, (in agreement with our study), under atropine, compared to control. Of note, the amount of atropine was lower in Ekblom et al (1972) study (2.0–2.5 mg), compared to the current one.

Contrary to our findings, Ekblom et al. (1972) and Davies et al. (1976) did not observe the modification of steady-state $\dot{V}$O_2_ during moderate exercise in upright posture, under atropine. Using normobaric hypoxia, which has been postulated to reduce vagal activity and increase sympathetic activity at rest, Lador et al (2008) observed that rest and exercise steady-state values for HR and CO were increased, without any change for $\dot{V}$O_2_.

## Conclusions

In conclusion, atropine administration suppresses *φ*_1_ for HR almost completely, in agreement with the vagal withdrawal concept at exercise onset. Therefore, only SV changes determine *A*_1_ for CO response after atropine injection. The differences in *A*_1_ for CO between atropine and control highlight the contribution of HR to the *A*_1_ for CO. These results provide the first direct demonstration of the effects of inhibition of vagal modulation of HR at exercise onset on the *φ*_1_ CO kinetics.

## Data Availability

The data that support the findings of this study are available from the corresponding author upon reasonable request.

## Abbreviations

*A*_1_: Amplitude of the first phase in double exponential model
*A*_2_: Amplitude of the second phase in double exponential model
ANOVA: Analysis of variance
CaO_2_–C$\overline{v }$O_2_: Arterial–venous O_2_ difference
CO: Cardiac output
CO_2_: Carbon dioxide
HR: Heart rate
[La]b: Blood lactate concentration
MAP: Mean arterial pressure
PCO_2_: Partial pressure of CO_2_
pH: Negative logarithm of hydrogen ion concentration
SV: Stroke volume
$\dot{V}$CO_2_: Carbon dioxide output
$$\dot{V}$$O_2_: Oxygen uptake
*φ*_1_: First phase of the double exponential model
*φ*_2_: Second phase of the double exponential model
*τ*: Time constant
